# Profile of sexuality in Moroccan chronic low back pain patients

**DOI:** 10.1186/1471-2474-14-63

**Published:** 2013-02-15

**Authors:** Hanane Bahouq, Allali Fadoua, Rkain Hanan, Hmamouchi Ihsane, Hajjaj-Hassouni Najia

**Affiliations:** 1 Department of Rheumatology, University Hospital El Ayachi, Rabat, Salé, Morocco; 2 Department of biostatistics, faculty of medicine and pharmacy of Rabat, Rabat, Morocco; 3 Department of physiology, faculty of medicine and pharmacy of Rabat, Rabat, Morocco

**Keywords:** Chronic low back pain, Sexuality, Sexual quality of life

## Abstract

**Background:**

Sexual life had an important role in preserving the good quality of life for patients and for their partner. Chronic Low Back Pain (CLBP) as other musculoskeletal diseases may affect all aspects of life including sexual functioning. The purpose of this study is to describe the impact of CLBP on the sexual life of patients and to identify the factors that affect their Sexual Quality of Life (SQOL).

**Methods:**

One hundred CLBP sexually active patients were included. Patients and disease Characteristics were collected. Impact on sexual life (sexual intercourse and SQOL) was also assessed. Univariate and multivariate analysis were performed to analyze significant determinants associated with the SQOL disturbance.

**Results:**

Eighty one percent of our patients complained about sexual difficulties related to CLBP. Libido decrease and painful intercourse position were reported respectively in 14.8 and 97.5% of cases. The most pain generating position was supine. Mean of sexual intercourse frequency decrease was at −10.4 ± 4.8 per month. SQOL score mean was at 44.6 ± 17.4%. Men suffered more than women from sexual problems (respectively 90% vs. 72%; p = 0.02). Men had worse SQOL than women (respectively 38.9 ± 17.2 vs. 50.3 ± 15.7%; p = 0.001). Univariate and multivariate analysis showed that advanced age (p = 0.009), poor functional status (p = 0.03), male gender (p = 0.03) and sexual intercourse frequency decrease (p = 0.005) were the independent variables associated with the SQOL disturbance.

**Conclusion:**

Our study suggests that sexuality is profoundly disturbed in CLBP patients; both their sexual intercourse and SQOL were affected. This disturbance seems to be associated with patient and disease characteristics. Sexuality should be taken into account in managing CLBP patients.

## Background

Chronic Low Back Pain (CLBP) is a current condition and a major public health problem, which causes considerable disability [1-3]. In addition to its functional and psychosocial impact, CLBP contributes to the decrease of sexual desire, sexual arousal and satisfaction [4,5]. Sexuality is a Multi-faceted complex that depends on anatomical, physiological, psychological, cultural and relational factors. A normal sexual function requires thus the integrity of the main systems of the body including the lumber and the muscular systems [1]. Few previous studies have evaluated the frequency and the importance of changes in the sexual desire or function in patients with CLBP [4]. Although the available literature recognizes sexuality as a very important factor in the quality of life and well being of patients and in spite of the fact that the sexual life is known to be highly disturbed in CLBP patients, data focusing on the CLBP impact on sexuality is still little investigated [4]. This insufficiency of attention to the sexual impairment related to CLBP may be due to a lack of education as well as to inhibitions of both patients and their doctors [4]. The purpose of this study is to contribute to the filling of this gap by describing the impact of the CLBP on the sexual life of patients and identifying eventual factors that are associated with their sexual quality of life (SQOL) disturbance.

## Methods

### Patients

We have included in this cross-sectional study, patients with CLBP seen in outpatient clinic at El Ayachi Hospital, Salé, Morocco. Informed consent was obtained from all patients and the National Research Ethics Committee approved the study. LBP was defined by pain, muscle tension, or stiffness localized below the costal margin and above the inferior gluteal folds, with or without leg pain (sciatica). Chronicity was established by the persistence of pain beyond 3 months of symptoms. We have included sexually active patients who are able to have erotic experiences and responses. Sexually active was defined by the ability to have sexual interest and to experience sexual attraction and intercourse by vaginal penetration. Were excluded all patients with no active sexual life and patients already operated or suffering from other comorbidities (diabetes, cardiovascular diseases, neurological problems, depression, sexual disorders as erectile dysfunction or anorgasmia) which could disturb sexuality.

After explaining the objectives of the study by the interviewer, the respondent was asked for his verbal consent to participate in the study. Only subjects who verbally consented were finally interviewed. One hundred forty patients, thus, were recruited, 15 patients were excluded and 25 patients refused to take part of this study.

### Methods

Because of the high percentage of illiteracy in our population, the study was conducted in the local Arabic language and the data was collected by a rheumatologist using a “face to face” interview with descriptive and explanatory structured questionnaire using specific and closed questions. The interviewer reads out the question from the questionnaire and the respondent provides the answer. The interview was anonymous and respected patient’s privacy and intimacy. We collected patients’ characteristics (age, educational level (analphabetic, university), Body mass index (BMI) and CLBP features (pain intensity assessed by the Visual Analogical Scale (VAS = 0–100 mm), disease duration and back pain disability assessed by the Arabic version of the Oswestry Low Back Pain Disability Questionnaire (Oswestry Disability Index: ODI) ranged from 0 to 100% with highest scores indicating maximum disability [6]). Then, we studied aspects of the CLBP impact on sexuality in all patients and for each gender. We assessed impact of the CLBP on sexual intercourse defined as all sexual acts contributing to a person bonding and considered intercourse with vaginal penetration (the decrease of libido was defined as a persistent or recurring inability to attain or maintain sufficient sexual excitement, causing personal distress, experienced as lack of subjective excitement, or lack of genital lubrication or other somatic responses; painful intercourse position defined as the most triggering back pain sexual positions and sexual intercourse frequency change because of the CLBP). To explore the change of the frequency of intercourse, we proceed by calculating the mean difference between the monthly intercourse frequency before and after low back pain onset. Question 8 of ODI was also used to assess sex life difficulties in our patients. In addition, we studied the sexual quality of life using the French version of the sexual quality of life questionnaire (SQOL - F [7] for women and the SQOL - M [8] for men). The SQOL-F has 18 items and the SQOL-M contains 11 items, each with a 6 point ranging from “completely agree” to “completely disagree”. Except for item 4 which corresponds to the only gender specific question, the male and female versions of the SQOL are very similar. The SQOL-M has seven fewer items than the original SQOL-F instrument: two on relationship; one related to emotional well-being; three related to frequency and avoidance of sexual activity; and one on the overall enjoyment. The items that have been removed in the SQOL-M are the ones that worked well for women, but did not work so well in a male population [8]. For example, questions on the sexuality-related emotional connection with the partner (e.g. “when I think about my sexual life I feel close to my partner”) may not resonate with men in the same way they do for women. Similarly, questions relating to avoidance and low frequency of the sexual activity because of sexual problems may be more relevant to the female sexual dysfunction than to the male sexual dysfunction [8]. The SQOL-F and SQOL-M are valid instruments for assessing the impact of the sexual dysfunction on the quality of life [7,8]. They showed good psychometric properties (convergent validity, discriminate validity and test-retest reliability) [7,8]. Total score of SQOL ranges from 0 to 100 [7,8]. Increasing scores employ better sexual quality of life. According to the data of the original validation study, men and women without a sexual dysfunction had high mean scores on the SQOL (87.13 and 90.1, respectively [7,8]).

Finally, we investigated factors, between patients and CLBP characteristics, affecting SQOL score.

### Statistical analysis

The Statistical Package for the Social Sciences software (SPSS, version 15, Chicago. Inc) was used for data processing and data analysis. Descriptive statistics included the median and quartiles, mean and standard deviation for interval variables; frequency and percentage for categorical variables. Comparisons between men and women were carried out by independent samples Student’s *t*-test for interval variables and the *χ*2 test for categorical variables. Univariate analysis was examined using Student’s *t*-test and correlations between SQOL score, patients, CLBP and sex life characteristics. Correlations were calculated with Spearman rank R. The remaining factors (P < 0.05) in the univariate analysis were entered into a multivariate linear regression model, so multivariate analysis were secondly performed to analyze significant determinants associated with SQOL disturbance using the SQOL score as dependent factors. ß Coefficient of each independent variable associated with altered SQOL score was defined. A P value of less than 0.05 was considered statistically significant.

## Results

Patients and CLBP characteristics are summarized in Table 1. Eighty one percent of our patients complained about sexual difficulties related to CLBP. Sex life was nearly normal but very painful, severely restricted and nearly absent because of pain in respectively 30, 18 and 11% of patients. Libido decrease and painful intercourse position were reported respectively in 14.8% and 97.5% of cases (Table 2). Supine was the most pain generating intercourse position followed by prone and side-lying positions (respectively 89.9, 24, 6.3%). Sexual intercourse frequency decreased by −10.40 ± 4.89 per month after chronic low back pain onset (mean intercourse frequency before CLBP 16.9 ± 4 per month vs. 6.5 ± 4.6 after CLBP onset; p < 0.0001). SQOL score mean was 44.6 ± 17.4% (Table 2).

**Table 1:** Chronic low back pain and patients characteristics

	**N = 100**
**Age (years)**	43.28 ± 7.5
**Male gender (%)**	50
**BMI (kg/m**^**2**^**)**	25.73 ± 5.1
**Educational level**	
**- Analphabetic (%)**	45
**- University (%)**	15
CLBP duration (in months)*	36 [24–72]
**VAS low back pain (0-100 mm)**	50 ± 20.7
**Oswestry (0-100%)**	41.6 ± 15.5

*Median and quartile, *BMI* Body Mass Index, *CLBP* Chronic Low Back Pain, *VAS* Visual Analogical Scale.

**Table 2:** Impact of chronic low back pain on patient’s sexual life

	**N = 100**	**Men N = 50**	**Women N = 50**	**p**
**Sexual problems (%)**	81	45 (90)	36 (72)	0.02
**Question 8 of Oswestry:**				
My sex life is normal and causes no extra pain (%).	13	8 (16)	5 (10)	0.4
My sex life is normal but causes some extra pain (%).	22	9 (18)	13 (26)	0.5
My sex life is nearly normal but is very painful (%).	36	17 (34)	19 (38)	0.9
My sex life is severely restricted by pain (%).	18	6 (12)	12 (24)	0.1
My sex life is nearly absent because of pain (%).	11	9 (18)	2 (4)	0.02
Pain prevents any sex life at all (%).	0			
**Sexual intercourse:**				
- Libido decrease (%)	12	2 (4)	10 (20)	0.01
- Painful intercourse position (%)	79	43 (86)	36 (72)	0.09
· Supine (%)	81	40 (80)	41 (82)	0.8
· Prone (%)	19	10 (20)	9 (18)	0.8
· Side-lying (%)	5	3 (8)	2 (4)	0.1
- Monthly intercourse frequency before CLBP onset	16.9 ± 4	17.7 ± 4.4	16.2 ± 3.7	0.04
- Monthly intercourse frequency after CLBP onset	6.5 ± 4.6	6.8 ± 4.3	6.3 ± 4.9	0.07
- Monthly intercourse frequency decrease	−10.40 ± 4.89	−10.9 ± 5.3	−9.9 ± 4.4	0.3
**Sexual quality of life:**				
- SQOL score (0- 100%)	44.65 ± 17.4	38.95 ± 17.2	50.35 ± 15.7	0.001

*CLBP* Chronic Low Back Pain, *SQOL* Sexual Quality Of Life.

Men suffered more than women from sexual problems (respectively 90% vs. 72%; p = 0.02). Sex life was nearly absent because of pain in women more than in men (25% vs. 4.4%; p = 0.01). Libido decrease was more reported by women (27.8% of women vs. 4.4% of men; p = 0.01) (Table 2). SQOL correlated significantly with age (r = −2.920; p = 0.003), disease duration (r = −0.266; p = 0.007), pain (r = −0.443; p < 0.0001), functional status (r = −0.214; p = 0.03) and sexual intercourse frequency decrease (r = −0.400; p < 0.0001). Men had worse SQOL than women (respectively 38.9 ± 17.2 vs. 50.3 ± 15.7%; p = 0.001). No relationship was found between SQOL, BMI, educational level, libido decrease, painful intercourse position and sex life difficulties assessed by question 8 of ODI (Table 3). In linear regression analysis, advanced age (ß = −0.54; p = 0.009), poor functional status (ß = −0.21; p = 0.03), male gender (ß = 7.6; p = 0.03) and sexual intercourse frequency decrease (ß = −0.90; p = 0.005) were the independent variables associated with SQOL disturbance (Table 4).

**Table 3:** Univariate analysis defining factors associated with sexual quality of life

	**R of Spearman**	**T student**	**p**
**Age**	−2.920		0.003
**Female gender**		50.3 ± 15.7	0.001
**Male gender**		38.9 ± 17.2	
**Analphabetic**		46.1 ± 16.8	0.4
**University level**		48.2 ± 21.5	
**BMI**	0.005		0.9
**Disease duration**	−0.266		0.007
**VAS low back pain**	−0.443		<0.0001
**Oswestry**	−0.214		0.03
**Question 8 of Oswestry (sex life)**	−0.095		0.34
**Decrease of libido**			
**No**		44.8 ± 17.6	0.7
**Yes**		43 ± 15.9	
**Painful intercourse position**			
**No**		45.2 ± 18.4	0.8
**Yes**		44.4 ± 17.2	
**Decrease of monthly intercourse frequency**	−0.400		<0.0001

*VAS* Visual Analogical Scale, *BMI* body mass index.

**Table 4:** Multiple linear regression analysis with sexual quality of life as dependent variable and patient and chronic low back pain characteristics as independent variables

	**ß (ES)**	**IC (95%)**	**P**
**Female gender**	1		
**Male gender**	7.6 (3.5)	(0.628-14.592)	0.03
**Age**	−0.54 (0.20)	(−0.945- 0.136)	0.009
**Disease duration**	−0.03 (0.027)	(−0.083- 0.023)	0.26
**VAS low back pain**	−1.63 (0.985)	(−3.59-0.32)	0.1
**Oswestry**	−0.21 (0.097)	(−0.405- 0.02)	0.03
**Monthly intercourse frequency decrease**	−0.90 (0.312)	(−1.526- 0.286)	0.005

*VAS* Visual Analogical Scale.

Adjusted R square = 0.342.

## Discussion

Our study shows that sexuality is disturbed in CLBP patients. Both sexual intercourse and SQOL were affected. This disturbance seems to be associated with patient and disease characteristics.

Sexual life had an important role in preserving the good quality of life for patients and for their partner [4]. CLBP as other musculoskeletal diseases may affect all aspects of life including sexual functioning [4]. The sexual act is a complex set of behaviors that involves most of the systems of the body as well as the low back. In consequence, any injury of the low back may interfere with sexuality [4]. Sexual difficulties in CLBP are frequent and ranging from 50% to 78% [4,9]. Surprisingly, this subject was very little investigated in CLBP patients. We found that 81% of our patients complain from sexual difficulties after the CLBP onset.

Various difficulties related to CLBP with respect to the sexual act [9-17] as well as the sexual quality of life were reported in literature [4]. Areas of arousal, positions, fear of exacerbating pain, low confidence, decreased frequency of sexual activity and complete cessation of sexual activity have been reported in literature [9-17]. Our patients have not only reported marked discomfort during intercourse and greater reduction in its frequency but also decreased sexual desire. These findings are similar to those previously reported by Maigne [4]. The effect of pain on the patient’s capacity for sexual intercourse is negative and may lead to disinterest and avoidance of any sexual activity [18]. There is a constant fear of exacerbating back pain by intercourse which may be responsible for the libido loss and for the decreased intercourse frequency [18]. In our study, lack of interest in sexual activity was more marked in women. Men and women are not equal regarding pain. Indeed, studies suggest that women suffer from pain more often and for a longer duration than man which may explain the marked libido loss face to an uncomfortable or painful intercourse in women [19].

Sexual intercourse positions usually adopted in our context are supine for women and prone for men [20]. These typical coital positions can create compressions and over extensions that trigger or aggravate pain in the lumber and interfere with any stage of the sexual response cycle [20]. Supine was the most painful intercourse position not only for women but also for men. This position was reported spontaneously by men as responsible of triggering and exacerbating back pain, so they avoid it during intercourse to make the experience less painful. Side-lying position is less commonly used in our society but it was the most comfortable. In our context, it’s extremely important to advice CLBP patients to adopt other uncommon intercourse positions in order to avoid pain, especially the side-lying position. The opposite was reported by Maigne, where supine was the most comfortable sexual intercourse position [4]. Divergence between these findings may be explained by the sociocultural context and sexual behaviors which are different between genders in Western and African societies.

The quality of sexual life can also be impaired by CLBP. The Quality of sexual life is not restricted to the physical sexual intercourse but it extends to a larger concept that encompasses the quality of all aspects of the sexual life. However, there are limited data using validated instruments on CLBP patients to assess this outcome measure. To our knowledge, only some studies have already explored SQOL in CLBP patients [4] and showed that CLBP is associated with a slightly greater reduction in the quality of sexual life [4,21].

As shown by SQOL score, the quality of sexual life in our patients was altered; it was more disturbed in older patients. There is a controversy about the relation between age and sexual activity in chronic pain patients as reported by previous studies [22-26]. In our society, no previous study was focused on this subject but it’s commonly admitted that older patients are less sexually active than younger persons [23,25].

Worse SQOL was identified in men comparing to women in our study. We thought that this difference of perceived sexual quality of life between men and women could be explained by social considerations. In fact, the sexual relationship is not equitable between women and men in our society. Difficulties in the sexual act are more negatively perceived by men, in whom, this could be responsible for a low self esteem and a virility injury. Comparatively to Maigne findings, sexual impairment was more marked in women and seems to be related to psychological factors [4]. This contradiction may be attributable to disparities on the perception of sexuality and response to pain [27] between men and women from different cultures.

In our sample, SQOL was more disturbed in patients with worst functional status. Prolonged disability may change patients’ feelings of sexual effectiveness and attractiveness to his or her partner, which may lead to a deficiency or a lack of sexual desire or sexual activity independently of pain intensity and disease duration [27]. According to our patients’ answers, we found that the sexual intercourse frequency decrease was related to the SQOL score more than to painful intercourse position and loss of libido. However, it appears extremely interesting to consider that perhaps loss of libido and painful intercourse are actually factors that may explain less frequent sexual activity.

Sexuality is not limited to sexual act but it also includes all emotional experiences in a sexual relationship [18,20]. In our society, there is an insufficiency of sexual education and a prematurity of sexual practice which can limit sexual blooming and restrict the sexual experience just to the level of the physical act [20].

Overweight was also reported as a factor associated with sexual problems in CLBP patients [28-31]. This factor wasn’t significantly associated to SQOL disturbance in our patients.

Some limitations should be pointed in our study. Firstly, data was collected by a “face to face” interview. This method permitted us to explain a question if a participant does not understand it and to collect detailed information. It also allowed more interaction between the respondents and the interviewers which can increase social desirability bias and fear of embarrassment. Self administered questionnaire is thus preferred in such sensible topic but it was limited in its use due to high non response. In fact, the effectiveness of a self administered questionnaire depends on the ability of the study population to read and comprehend the questions. However, because of the high percentage of illiteracy in our population, this method could not be envisaged in our context. Secondly, sexual activity is a very large concept and very a difficult one to define. It should be emphasized that physicians who deal with low back pain must be prepared and formed to deal with all aspects of their patient's sexual life difficulties. Finally, a sexologist participation in this study would be very interesting in broaching this delicate subject. It seems to be very useful to cooperate with the sexologist to manage sexual difficulties in CLBP patients. Further studies are clearly warranted and should include other facets of this subject, particularly psychosocial factors and partner’s experiences.

## Conclusion

Sexuality is negatively affected in patients suffering from CLBP. Disturbances affected the sexual act as well as the sexual quality of life. Many factors are associated with these disturbances. Altered sexual quality of life was relatively more common in men and older patients and was due to sexual intercourse frequency decrease rather than to back pain and libido loss. This effect on the sexual quality of life was also associated with poor functional status. It appears clearly that sexuality management must be integrated into CLBP patients care and factors associated with it should be considered in future interventions to positively promote the sexual function in CLBP patients.

## Abbreviations

CLBP: Chronic low back pain; SQOL: Sexual quality of life; VAS: Visual analogical scale; BMI: Body mass index; SPSS: Social sciences software.

## Competing interests

The authors declare that they have no competing interests.

## Authors’ contributions

Dr. HB carried out the design of the study, collected the data and performed the statistical analysis. Dr. HR and Dr. IH made substantial contributions to the conception, design, analysis and interpretation of data. Dr. FA revised the manuscript and gave final approval of the version to be published. Dr. NHH supervised the collection of data and the research group work. All authors read and approved the final manuscript.

## Pre-publication history

The pre-publication history for this paper can be accessed here:

http://www.biomedcentral.com/1471-2474/14/63/prepub
